# Analysis of global stock index data during crisis period via complex network approach

**DOI:** 10.1371/journal.pone.0200600

**Published:** 2018-07-18

**Authors:** Bentian Li, Dechang Pi

**Affiliations:** 1 College of Computer Science and Technology, Nanjing University of Aeronautics and Astronautics, Nanjing, China; 2 Collaborative Innovation Center of Novel Software Technology and Industrialization, Nanjing, China; Universidad Veracruzana, MEXICO

## Abstract

Considerable research has been done on the complex stock market, however, there is very little systematic work on the impact of crisis on global stock markets. For filling in these gaps, we propose a complex network method, which analyzes the effects of the 2008 global financial crisis on global main stock index from 2005 to 2010. Firstly, we construct three weighted networks. The physics-derived technique of minimum spanning tree is utilized to investigate the networks of three stages. Regional clustering is found in each network. Secondly, we construct three average threshold networks and find the small-world property in the network before and during the crisis. Finally, the dynamical change of the network community structure is deeply analyzed with different threshold. The result indicates that for large thresholds, the network before and after the crisis has a significant community structure. Though this analysis, it would be helpful to investors for making decisions regarding their portfolios or to regulators for monitoring the key nodes to ensure the overall stability of the global stock market.

## 1. Introduction

The global stock market is a typical complex system. The complex network is a powerful tool to deal with complex system [1–4]. Originally, stock market research focuses on different countries, including the works of Onnela JP et al. [5] for New York stock exchange, Huang WQ et al. [6], Qiao H et al. [7] and Long H et al. [8] for Chinese stock market, Vizgunov A et al. [9] for Russian stock market, Namaki A et al. [10] for Tehran stock exchange of Iran, Radhakrishnan S et al. [11] for Thailand SET index, Birch J et al. [12] for German DAX30 index. Zhao L et al. [13] for S&P 500 constituent stocks. It is found that there are many rules and characteristics in the financial network, such as the topological properties, the faction structure and scale-free characteristic. The discovery of these rules is of great value to the healthy development of stock market and the risk control of investors.

In the past ten years, it is worth noting that the study of relationship between the financial crisis and the stock market structure has gained more and more attention [14–17], because the financial crisis is a destructive economic phenomenon for global stock market. Typically, Kantar E et al.[18] analyzed the topology structure and clustering structure of 50 important Turkey Company[18] using the method of minimum spanning tree (MST). The study found that Turkey's company is less affected by the financial crisis. Yang R et al.[19] also uses the minimum spanning tree (MST) and hierarchical tree to study the network of China International Trust and Investment Corporation (CITIC) industrial index from 2006 to 2013. The results showed that there exists core node and the obvious industrial cluster in the network, and found that the degree of clustering is enhanced in a smaller range during the financial crisis. Zhao L et al. [13] studied the S&P500 stock based on correlation method[20]. The study confirmed that the network has global expansion and local aggregation behavior during the crisis. Nobi A et al. [21] studied the change of the network structure in 2000–2012 based on the correlation between the global index and the Korean local index. It was found that the dynamic change of the Jaccard similarity index[22] could be used as the index of systemic risk or crisis. Yan XG et al.[23] analyzed the closing price data of 710 stocks of Shanghai Stock Exchange (SSE) from 2005 to 2011, and divided the time segments into three sub-stages according to the occurrence time of US sub-prime crisis. The stability and robustness of the network are investigated. Recently, Cao G et al. [24] analyzed the whole and partial characteristics of international stock market network. For the whole network, the mechanism of robustness was analyzed. For the partial network, the dynamic evolution of the relationship between the Chinese (Shanghai) stock market and the international stock market was explored using the sliding window method[25]. Considerable research has been done on the complex stock market, however, there is still very little systematic work on the impact of crisis on global stock markets.

In summary, we find that researchers mostly focused on specific native stock markets, such as US stock market, Korean stock market and Chinese stock market. The effect of financial crisis on the country's stock network was studied separately. There is little research on national stock data from a large system perspective. For filling in these gaps, we propose a complex network method, which analyzes the effects of the 2008 global financial crisis on global main stock index from 2005 to 2010. Three weighted networks and three threshold networks are constructed. Firstly, the physics-derived technique of minimum spanning tree is utilized to investigate the taxonomy information of three weighted networks. Secondly, we explore the small-world property of the average threshold network by the way of clustering coefficient and characteristic path length. Thirdly, we also further deeply analyze the change of community structure of threshold networks with different thresholds. These research will give a helpful exploration to uncover the mechanism of effect of global financial risks on the global stock market.

The rest of this paper is organized as follows: Section 2 introduces the research method, including data processing and constructing the network, network parameter calculation. Section 3 is the part of empirical analysis and discussion. Finally, the summary and conclusion are given in Section 4.

## 2. Research methods

### 2.1 Data processing

**Fig 1:**
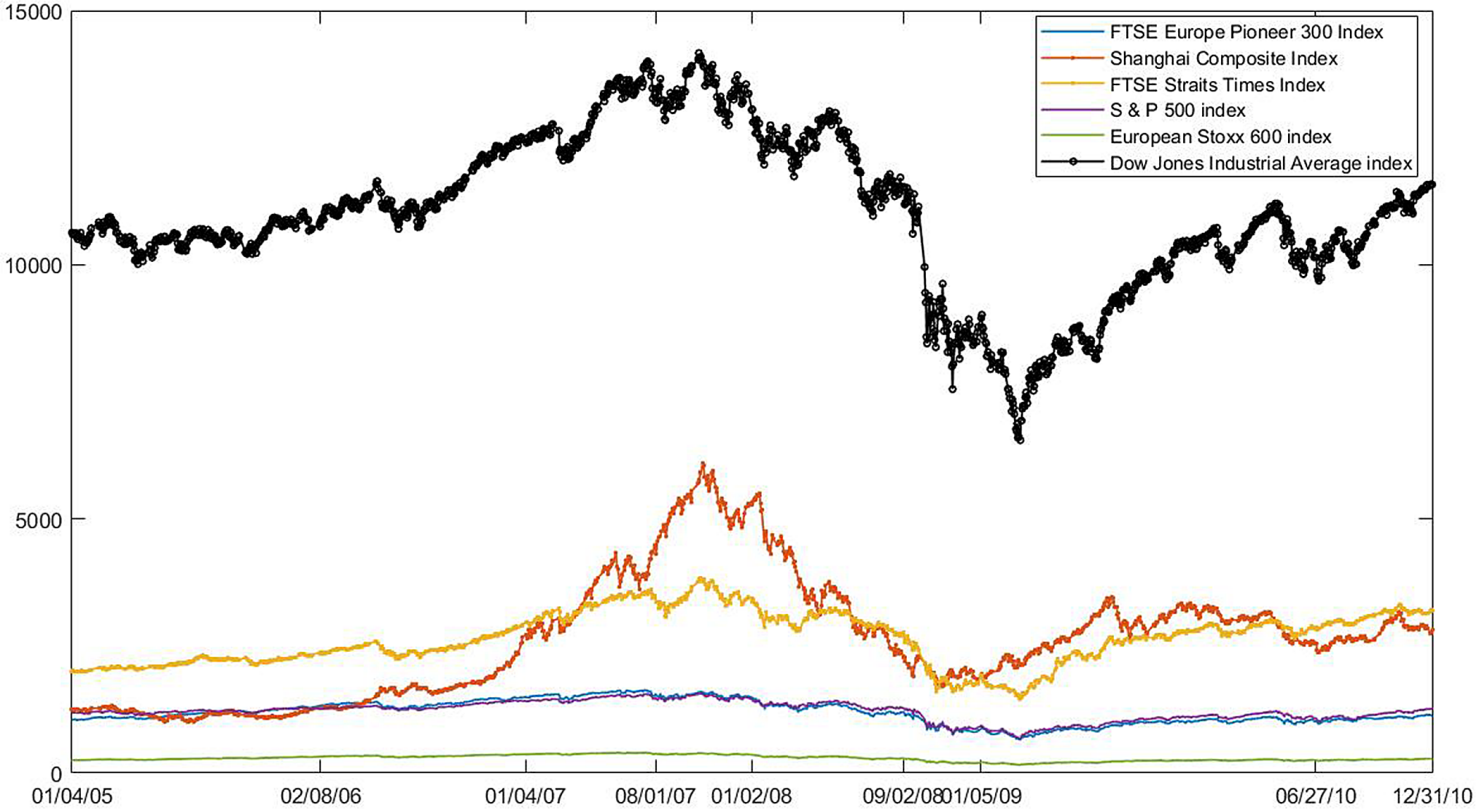
Volatility of the global representative stock index: 2005–2010. The volatility of the global representative stock index from the year 2005 to 2010. They are FTSE Europe Pioneer 300 Index, Shanghai Composite Index, FTSE Straits Times Index, S & P 500 index, European Stoxx 600 index and Dow Jones Industrial Average index.

The 2008 financial crisis originated in the United States and has caused a global stock market crash. The volatility curve of the global representative stock index from 2005 to 2010 is shown as Fig 1. They are FTSE Europe Pioneer 300 Index, Shanghai Composite Index, FTSE Straits Times Index, S & P 500 index, European Stoxx 600 index and Dow Jones Industrial Average index. However, as is well known to all, the evolution of the subprime mortgage crisis created a financial crisis. This means that there is a certain incubation period before and after the outbreak. Like the previous work, Kantar E et al. [18] thinks 2008 as the crisis time and Zhao L et al. [13] thinks that subprime mortgage crisis is from 2007 to 2009 and Nobi A et al. [21] considers that the mortgage crisis is in 2007 and global financial crisis is in 2008. That is to say, it is generally believed that the American subprime crisis has triggered a global economic crisis. Besides, in this paper, the crisis refers to the subprime crisis and the accompanying financial turmoil. The differences and synchronization of the stock index in different country should be considered at the same time. So, we eventually set the period from 4 January 2007 to 31 December 2008 as financial crisis time. 38 publicly traded stock indexes collected from Yahoo! Finance[26] are selected during the five years, which is shown as S1 File, S2 File and Table 1. According to the financial crisis time, the stock data is divided into three periods. The stage before the crisis is from 4 January 2005 to 29 December 2006. The stage during the crisis is from 4 January 2007 to 31 December 2008. The stage after the crisis is from 5 January 2009 to 31 December 2010. Then, the data of non-trading days and no transaction on the same day is removed. At last we got stock intersection data of three periods, including 315 trading days in the stage before crisis and 311 trading days in the stage during crisis and 144 trading days in the stage after crisis.

**Table 1:** 38 Global main stock indexes. This is the name of the 38 global main stock indexes in the world. We collected from Yahoo! Finance. For the convenience of expression and computation later, we numbered it. For each item, the front is its serial number, followed by the corresponding stock index.

Number. Index	Number. Index
1. FTSE Europe Pioneer 300 Index	20. Belgium 20 index
2. TSX composite index	21. France CAC40 index
3. Shanghai Composite Index	22. Baltic BDI Index
4. Denmark Copenhagen 20 index	23. Thailand SET index
5. Russia RTS index	24. Shenzhen Component Index
6. Iceland stock index	25. Australian Common Stock Index
7. Mumbai India SENSEX30 index	26. Irish Composite Index
8. Austria ATX index	27. Sweden Stockholm 30 Index
9. FTSE Straits Times Index	28. Switzerland SMI index
10. FTSE Malaysia KLCI Index	29. NASDAQ Composite Index
11. Brazil IBOVESPA stock index	30. Dollar index
12. Greece Athens ASE Index	31. Finland Helsinki 25 Index
13. German DAX30 index	32. UK FTSE 100 Index
14. Italy FTSE MIB Index	33. Netherlands Amsterdam Index
15. New Zealand 50 Index	34. Philippine Securities Index
16. Nikkei 225 Index	35. Portugal PSI20 index
17. S & P 500 index	36. Spain IBEX35 index
18. S & P Australia 200 Index	37. Dow Jones Industrial Average index
19. European Stoxx 600 index	38. Korea KOSPI Index

### 2.2 Constructing network

Let *p*_*i*_(t) represents the closing price of a stock index *i* at day *t*. The return of logarithmic rate from *t*−Δ*t* to *t* is defined as:

$$R_{i}(t)=\ln p_{i}(t)-\ln p_{i}(t-\Delta t)$$ (1)

Where Δ*t* is the time interval. Like the Huang WQ et al. [6] and Zhao L et al. [13], we also take Δ*t* as one day in the following analysis throughout this paper. The Pearson correlation coefficient of return rate between stock index *i* and *j* is defined as:

$$\rho_{ij}=\frac{E(R_{i}R_{j})-E(R_{i})E(R_{j})}{\sqrt{Var(R_{i})Var(R_{j})}}$$ (2)

*E*(*R*_*i*_) is the average rate of return during the period *n* of stock index *i*, which is defined as:

$$E(R_{i})=\frac{1}{n}\sum_{t=1}^{n}R_{i}(t)$$ (3)

**Fig 2:**
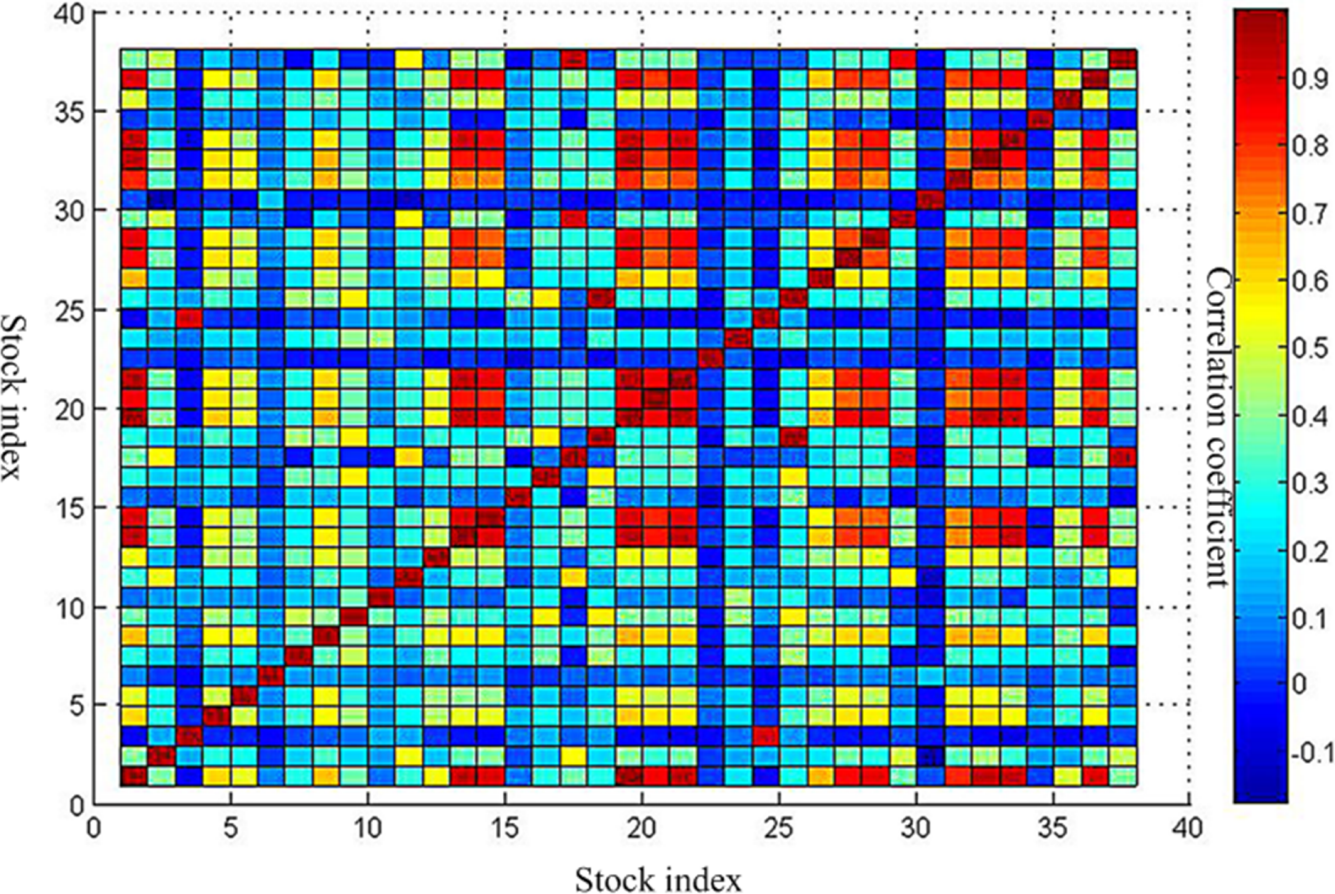
Correlation coefficient matrix before the crisis. This is the correlation coefficient matrix before the crisis. This is corresponding to data in S3 File. The vertical axis and the horizontal axis all represent the 38 stock indexes. The redder the color of the block area, the greater the correlation coefficient. The bluer the color of the block area is, the smaller the correlation coefficient is.

**Fig 3:**
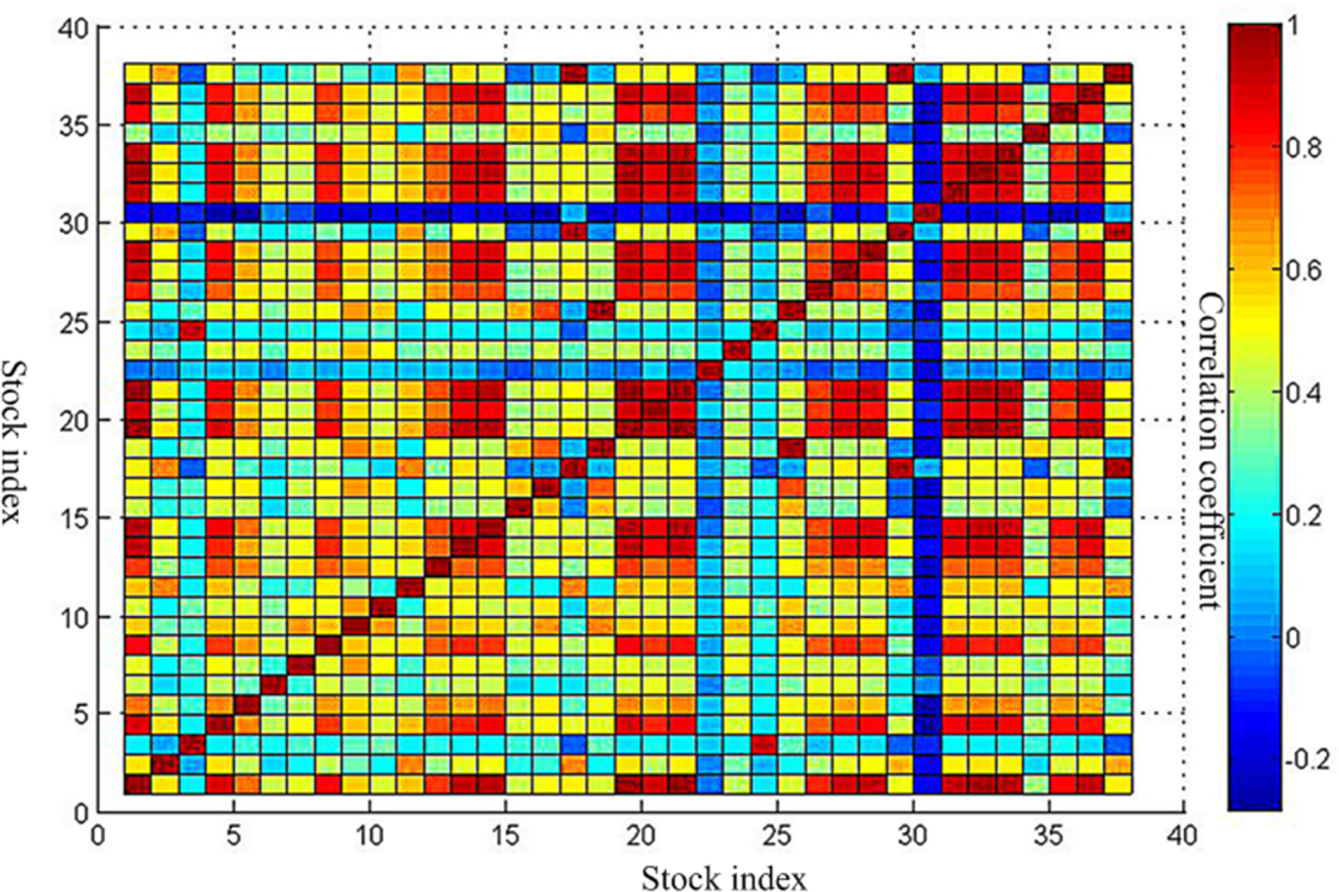
Correlation coefficient matrix during the crisis. This is the correlation coefficient matrix during the crisis. This is corresponding to data in S3 File. The vertical axis and the horizontal axis all represent the 38 stock indexes. The redder the color of the block area, the greater the correlation coefficient. The bluer the color of the block area is, the smaller the correlation coefficient is.

**Fig 4:**
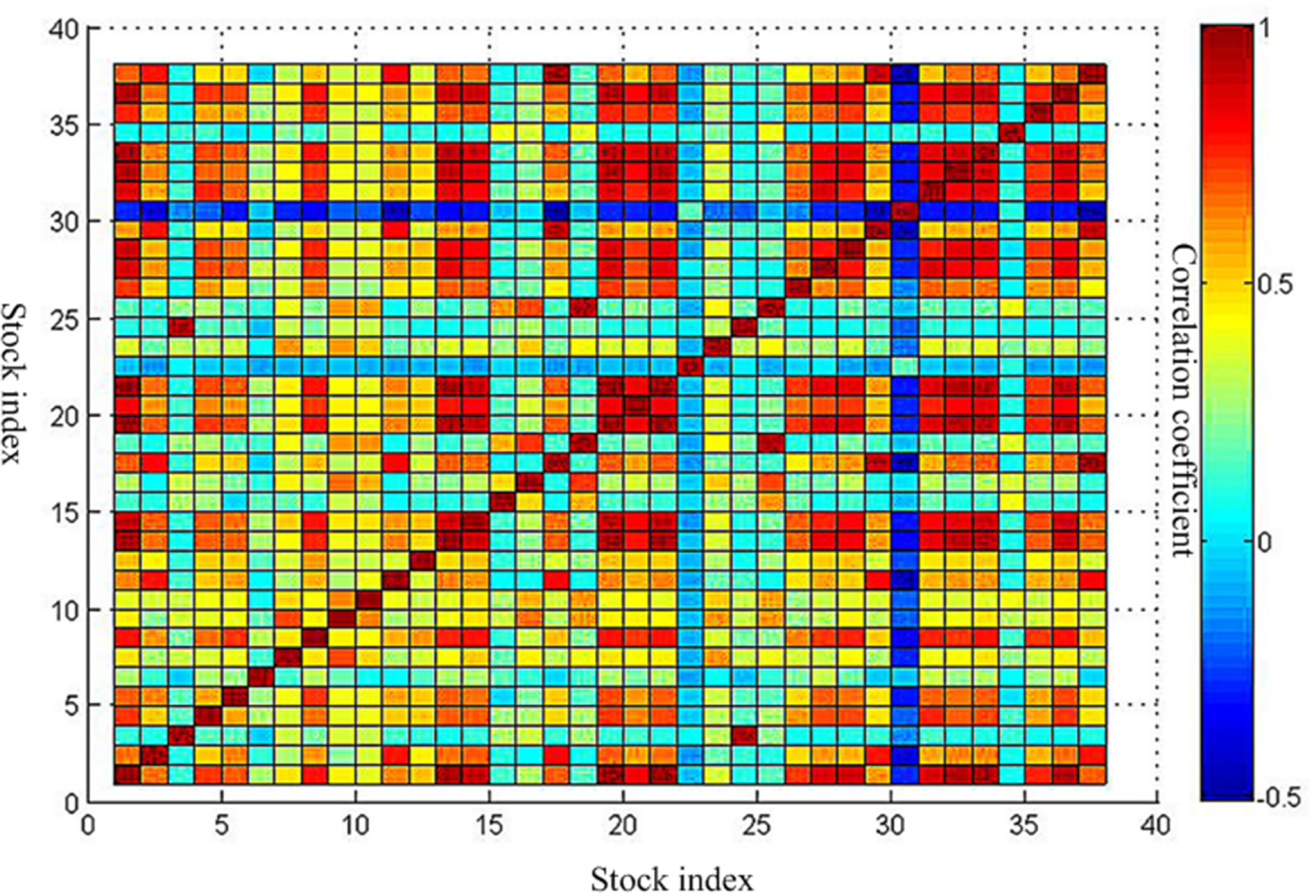
Correlation coefficient matrix after the crisis. This is the correlation coefficient matrix after the crisis. This is corresponding to data in S3 File. The vertical axis and the horizontal axis all represent the 38 stock indexes. The redder the color of the block area, the greater the correlation coefficient. The bluer the color of the block area is, the smaller the correlation coefficient is.

Three periods of the correlation coefficient matrix are shown in S3 File and Figs 2–4.

#### 2.2.1 Three weighted networks

Considering that correlation coefficient cannot be used to as a distance between the two stocks, we transform the correlation coefficient to distance, which is proposed by Mantegna RN[27]. An appropriate function for this transformation is defined as

$$d_{ij}=\sqrt{2(1-\rho_{ij})}$$ (4)

So, we use this definition to define the distance of a pairs of stocks. The distance between stocks is regarded as a weight in the network. Three weighted network could be constructed in this way.

#### 2.2.2 Three threshold networks

For constructing the threshold network, it is important to note that the selection of the threshold defined by *θ*. It is worth noting that different thresholds may have diverse effects. Similar to the previous work studied by Huang WQ et al. [6] and Zhao L et al. [13], we first choose the average value of the correlation coefficient as threshold *θ* through many experiments for exploring the small-world property of the network. Then we also analyzed the impact of dynamic thresholds on the network community structure.

After the threshold *θ* is selected, any two stock index *i* and *j* is treated as a node in the network. Then, if the correlation coefficient *ρ*_ij_ is greater than or equal to the specified threshold *θ*, the link between node pair is considered to exist, otherwise it does not exist. Besides, it is assumed that the link do not have direction and the weight coefficient is 1, where *ρ*_*ij*_,*θ*∈[−1,1]. In this method, three threshold networks are established which is before, during and after the crisis.

### 2.3 Network parameters

#### 2.3.1 Average threshold

$$\theta =\frac{1}{m}\sum_{i=1}^{m}\rho_{i}$$ (5)

*ρ* denotes correlation coefficient. *m* represents the number of combinations between any two different stocks. This parameter represents the average degree of interconnection of network nodes.

#### 2.3.2 Clustering coefficient

For undirected network, given *i* node, *k*_*i*_ represents the number of adjacent nodes. The maximum number of possible edges for the *k*_*i*_ is *k*_*i*_(*k*_*i*_−1)/2. The actual number of edges is *E*_*i*_. So the clustering coefficient for node *i* is defined as.

$$C_{i}=\frac{2E_{i}}{k_{i}(k_{i}-1)}$$ (6)

So, the clustering coefficient of the whole network could be obtained by averaging the clustering coefficients of all nodes. It used to describe the aggregation of the entire network. For a total of *N* nodes in the network, the formula is defined as follows.

$$C=\frac{1}{N}\sum_{i=1}^{N}C_{i}$$ (7)

#### 2.3.3 Characteristic path length

For the network, the characteristic path length is defined as the average of the distance between two and two nodes all of network, which is defined as follows.

$$L=\frac{1}{\frac{1}{2}N(N+1)}\sum_{i>j}d_{ij}$$ (8)

Among them, *d*_*ij*_ is the distance between nodes, that is, the minimum number of edges between any two nodes. *N* denotes the total number of network nodes. Characteristic path length represents the cost of separating two nodes in a network. Take social networks as an example, there are a large number of people in the network as nodes, but the average path of the network is very small. This shows that any two people need to know each other only through a small amount of middleman introduction. This is the so-called small world phenomenon[28].

## 3. Empirical analysis and discussion

### 3.1 MST analysis

**Fig 5:**
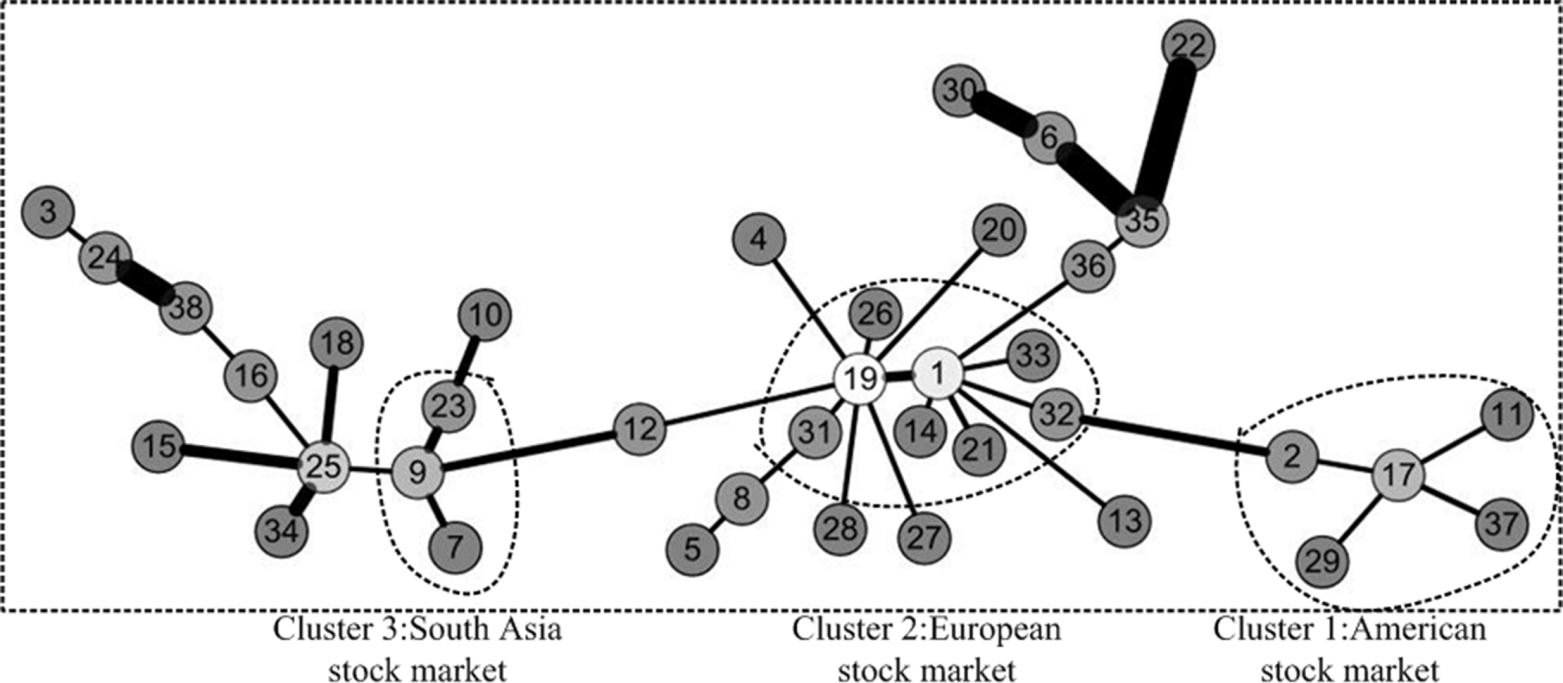
Minimum spanning tree of network before the crisis. The network before the crisis is divided into three areas of aggregation. They are American stock market, Asia Pacific& Australia stock market and European stock market. The lighter the color of the node is, the greater the degree of the node is. In addition, the thicker the edge is, the greater the weight is in the figure.

**Fig 6:**
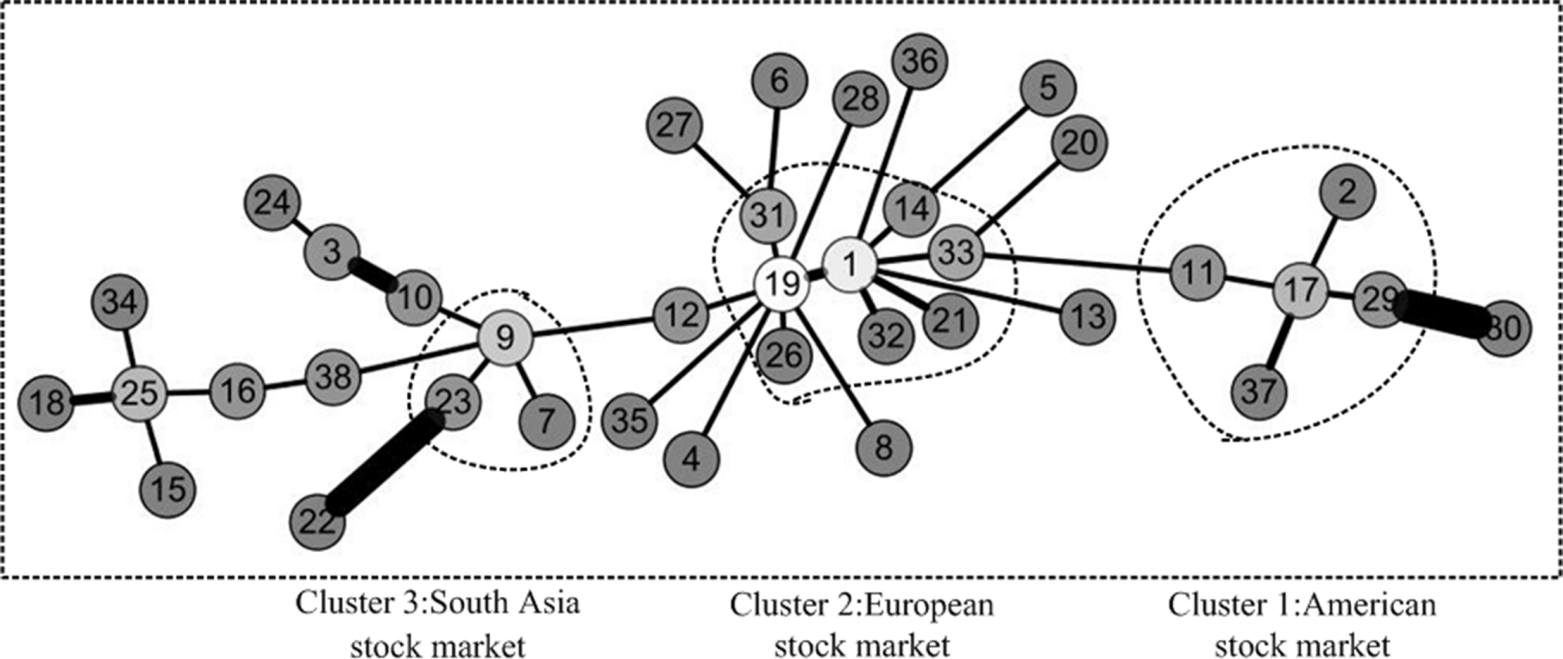
Minimum spanning tree of network during the crisis. The network during the crisis is divided into three areas of aggregation. They are American stock market, Asia Pacific& Australia stock market and European stock market. The lighter the color of the node is, the greater the degree of the node is. In addition, the thicker the edge is, the greater the weight is in the figure.

**Fig 7:**
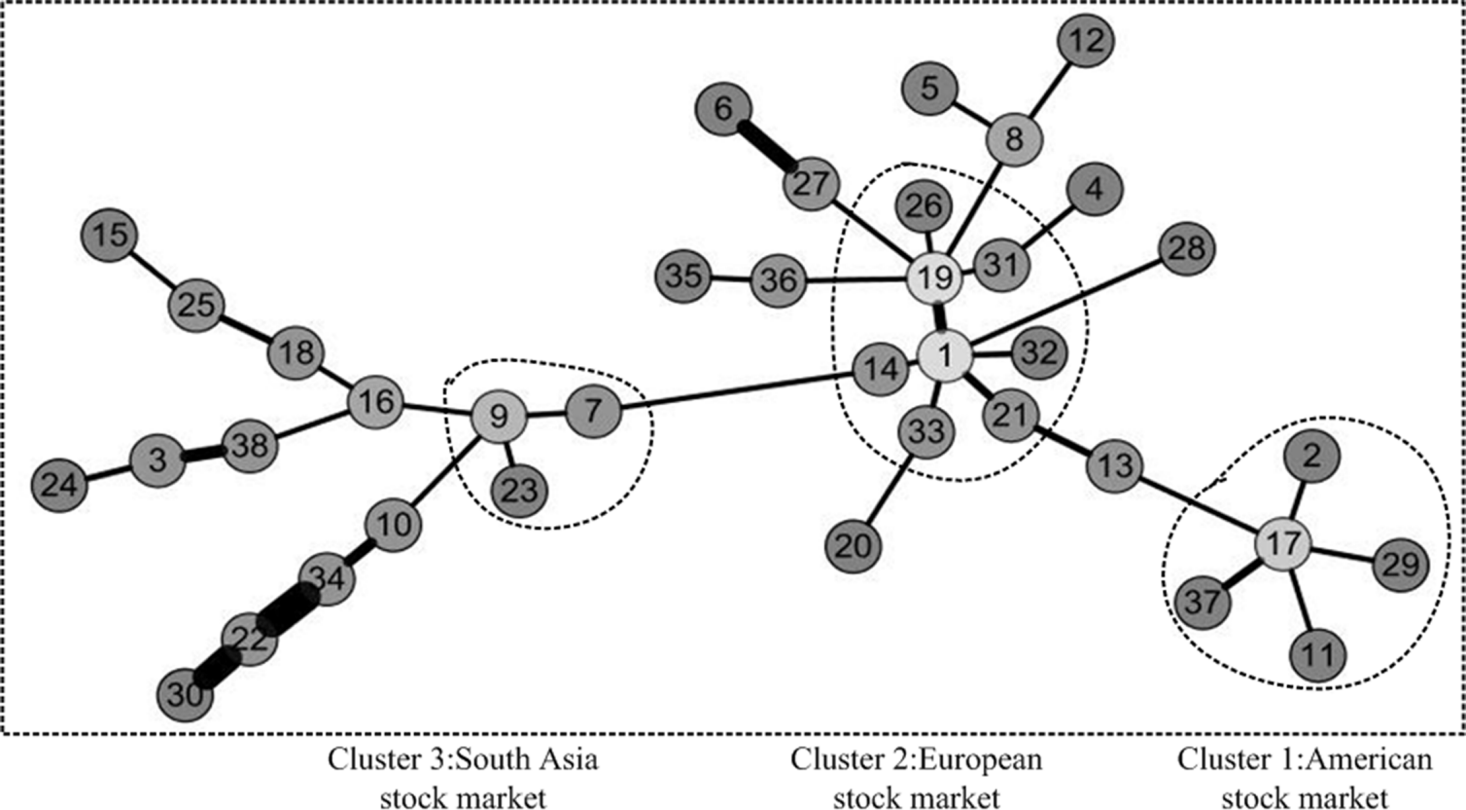
Minimum spanning tree of network after the crisis. The network after the crisis is divided into three areas of aggregation. They are American stock market, Asia Pacific& Australia stock market and European stock market. The lighter the color of the node is, the greater the degree of the node is. In addition, the thicker the edge is, the greater the weight is in the figure.

The method for constructing minimum spanning tree(MST) was proposed by Kruskal JB [29]. It is referred to as the Kruskals algorithm. It is used first to extract taxonomy information from the economic data of financial market by Mantegna RN [27]. Inspired by the above application, in this paper, we also use this method to analyze for discovering clustering phenomena in three weighted networks. We first take the distance of a pair of stocks as the weight of network. Then, the minimum spanning tree of three periods of network was constructed, which is shown as Figs 5–7. It is worth noting that the lighter the color of the node is, the greater the degree of the node is. Besides, the thicker the edge is, the greater the weight is in the figure. We have adopted a similar approach to Literature Yang R et al.[19] and Kantar E et al.[18].

Through a comparative analysis of three periods of minimum spanning tree of network, Firstly, the obvious clusters with at least three nodes could be founded in all network, which means these nodes in the clusters have a particularly stable relationship in all three periods of the network. For instance, the first cluster consists of 11 Brazil IBOVESPA stock index, 2 TSX composite index, 29 NASDAQ Composite Index, 17 S & P 500 index and 37 Dow Jones Industrial Average index. The above index is all from American stock market. These index remain clustered together all the time, meaning that these five indexes have kept a close relationship. This is probably because they are from Brazil, Canada and the United States, which are the three largest economic entity of the Americas, leading to closer ties in the financial stock market. The second cluster consists of 26 Irish Composite Index, 19 European Stoxx 600 index, 21 France CAC40 index, 33 Netherlands Amsterdam Index, 31 Finland Helsinki 25 Index, 1 FTSE Europe Pioneer 300 Index, 14 Italy FTSE MIB Index, 32 UK FTSE 100 Index. The above index is all from European stock market. These stock indices are all from European Union Member States, which maintain stable dependence and association with each other. The third cluster consists of 23 Thailand SET index, 9 FTSE Straits Times Index, 7 Mumbai India SENSEX30 index. The above index is all from Southeast Asia stock market. They come from Thailand, Singapore and India, which are the member of the Association of Southeast Asian Nations. So, the economic and financial markets have maintained a stable relationship. We could also see from above Figs 2–4 that the correlation coefficients between stock indices in each clusters are relatively high. Therefore, these stock indexes are strongly connected with each other. Secondly, the change of weight between stock indexes is not balanced. Some stock indexes maintain a stable weight connection in three weighted network, such as the number index of 1 and 19, the number index of 3 and 24, and the number index of 17, 2, 29, 37, 11. This is because they come from the same country or cluster. Other stock indices vary greatly in their weight. The typical representative is that as the source of the crisis, the US stock market fluctuated at the earliest, which the NASDAQ Composite Index and the Dollar index have the greatest weight in Fig 6. However, in the other two Figs 5 and 7, they did not show such a big weight. Thirdly, we analyze the changes in the number of edges per node before and after the crisis. The core node number 19 with 8 edges before the crisis and the core node number 19 with 6 edges after the crisis. It indicated that the financial crisis indeed had impacted the indexes network. This may be related to the global market downturn after the crisis.

### 3.2 Small-world property

Base on the average threshold network for three periods, we first got the static network topology characteristic parameter including the average threshold, number of nodes, number of edges, clustering coefficient and characteristic path length for further analysis, which is shown as Table 2. Comparing the network constructed by the specific average threshold of three periods, we could find that average threshold during the crisis does have the highest value. It indicates that the global main stock index is closely linked in this stage. Besides, the value of the clustering coefficient and characteristic path length of the threshold network during the crisis is less than other periods.

**Table 2:** Network topology characteristic parameter of three periods. These parameters include average threshold, number of nodes, number of edges, clustering coefficient and characteristic path length for three different periods.

Period	Average threshold	Node	Edge	C	L
**Before Crisis**	0.281	35	315	0.787	1.456
**During Crisis**	0.438	36	359	0.780	1.381
**After Crisis**	0.370	35	332	0.817	1.724

Meanwhile, this paper focuses on the small world characteristics of the global stock index network from the view of clustering coefficient and characteristic path length comparing with the random network with the same scale. Generally speaking, a small-world network is characterized by a ratio gamma *γ* between the clustering-coefficient *C* of the specific network and the clustering-coefficient *C*_*random*_ of a random graph of >1 and a ratio lambda *λ* between the path length *L* of the specific network and the path length *L*_*random*_ of a random graph of ≈1. The small-world-ness of a graph can be expressed in a single parameter sigma *σ*, defined as the ratio between gamma and lambda. Sigma is typically >1 for networks with a small-world organization[30,31]. Specifically speaking, the definition are as follows.

$$\gamma =\frac{C}{C_{random}}$$ (9)

$$\lambda =\frac{L}{L_{random}}$$ (10)

$$\sigma =\frac{\gamma }{\lambda }$$ (11)

Through further analysis, we could find the value of gamma, lambda and sigma, which is shown as Table 3. It's not hard to see the value of gamma and sigma meet the conditions >1 in all three threshold networks. Besides, the value of lambda in the stage of before the crisis and during the crisis meet the above conditions ≈1. These results mean the network before the crisis and during the crisis exists small world characteristics evidently.

**Table 3:** Small world characteristic statistical parameter. These parameters include ratio gamma *γ*, ratio lambda *λ*, sigma σ, clustering coefficient and characteristic path length in the random network with the same scale.

Period	*C*_*random*_	*L*_*random*_	γ	λ	σ
**Before Crisis**	0.581	1.470	1.354	0.990	1.367
**During Crisis**	0.618	1.430	1.262	0.966	1.306
**After Crisis**	0.576	1.442	1.418	1.195	1.186

Especially, among them, we also focus on the value of sigma, which could be used as an indicator of comparative ‘small-world-ness’[30]. It is known from the result the value of *σ* before the crisis is the largest. In the other two periods, the value gradually decreases. This suggests a strong aggregation properties and relations of the stock market before the crisis. So, when the crisis broke out, the stock market crashed collectively. For the network during the crisis, we could also conclude that the stock market has the small world characteristics. From the smaller average path, this shows that during the financial crisis, the changes of one country's stock index could easily affect another country's stock index. From the larger clustering coefficient, it shows that the spread of a certain range of price volatility during the crisis is rapid, which reflects the synchronization of the fluctuation of the stock market in a certain extent. For the network after the crisis, the value of *σ* is the smallest. This could suggest that the interconnection of the global stock market is weakening after the crisis.

### 3.3 Dynamic changes of community structure

**Fig 8:**
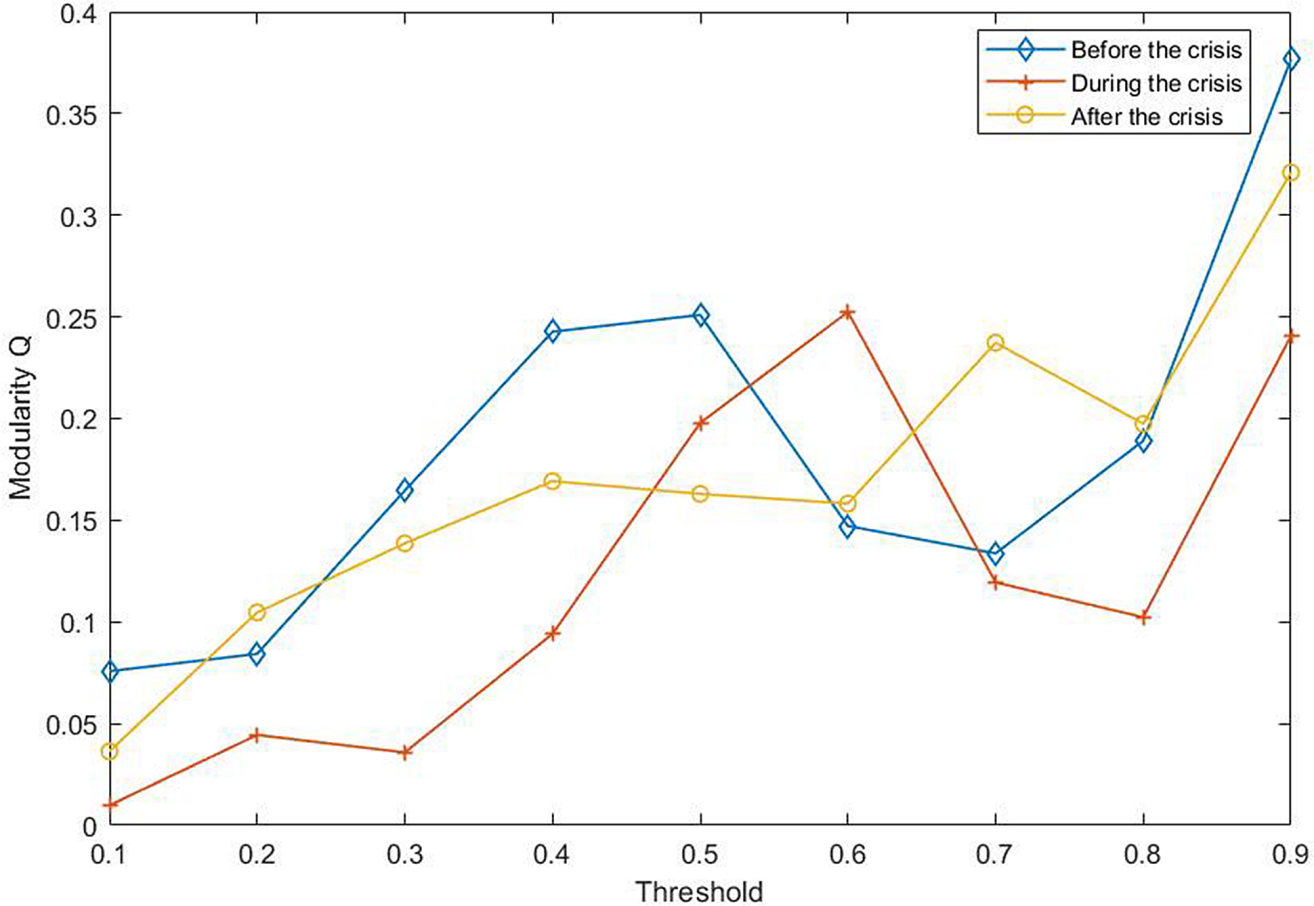
The influence of the threshold on the modularity Q. This is the modularity variation curve of the network in three different periods under different threshold conditions. The values greater than about 0.3 appear to indicate significant community structure.

Considering the different effects of threshold selection on the network, we first analyze the effect of threshold on the network modularity Q, as showed in Fig 8. In view of the definition of the structure of the community, if a particular division gives no more within-community edges than would be expected by random chance we will get Q = 0 [32]. Values other than 0 indicate deviations from randomness, and in practice values greater than about 0.3 appear to indicate significant community structure [32]. As the result shows, when the threshold is 0.9, the network before and after the crisis has a more obvious community structure. The middle of the crisis is the opposite. Besides, it shows that the community structure of the network has a change from obvious to weak, and then from weak to obvious in this threshold.

**Fig 9:**
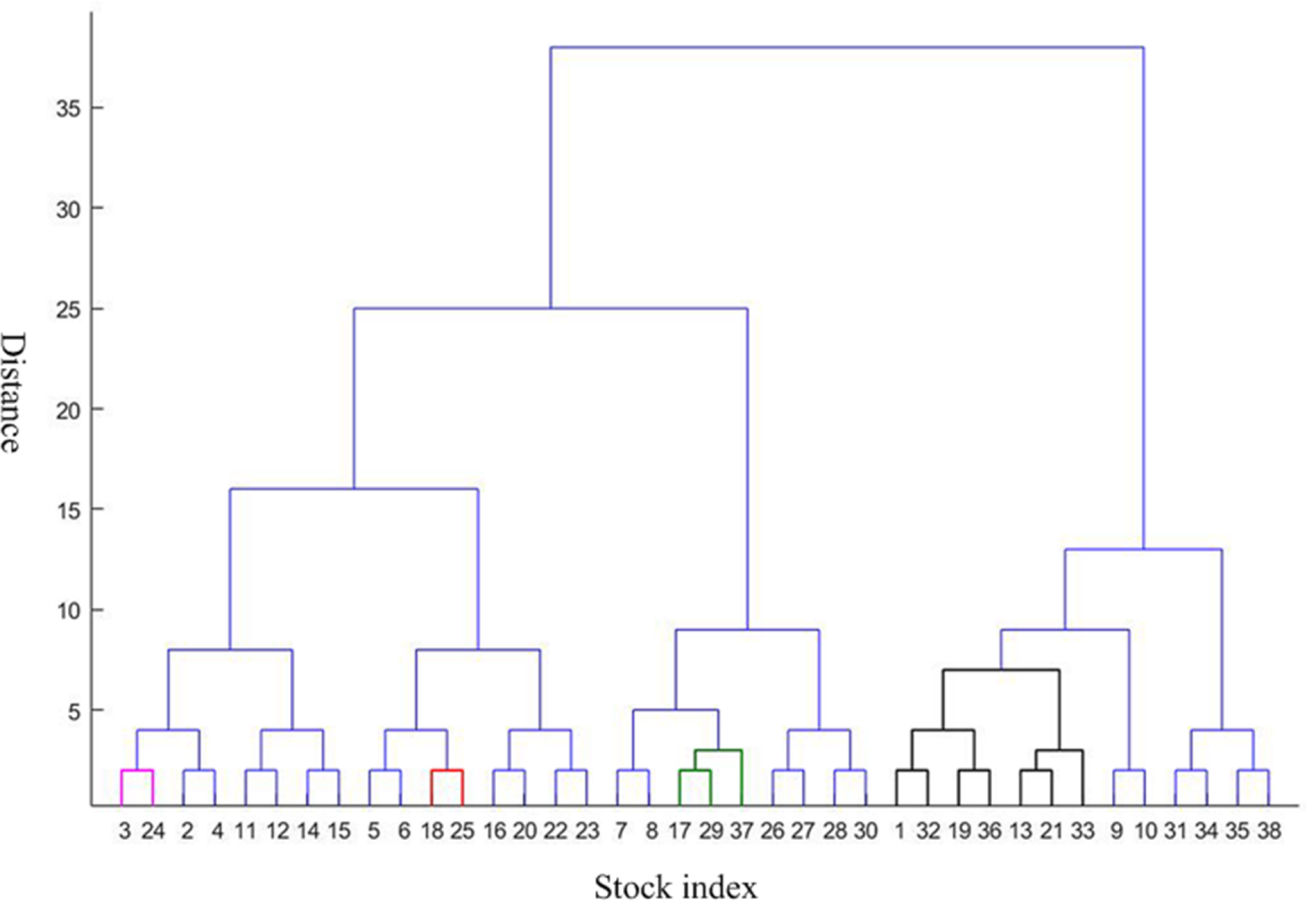
Hierarchical clustering structure before the crisis. This is the hierarchical clustering structure before the crisis. The horizontal axis represents the stock index, while the vertical axis represents the distance between these indices. The network could be divided into four communities whose member is shown in purple, red, green and black line.

**Fig 10:**
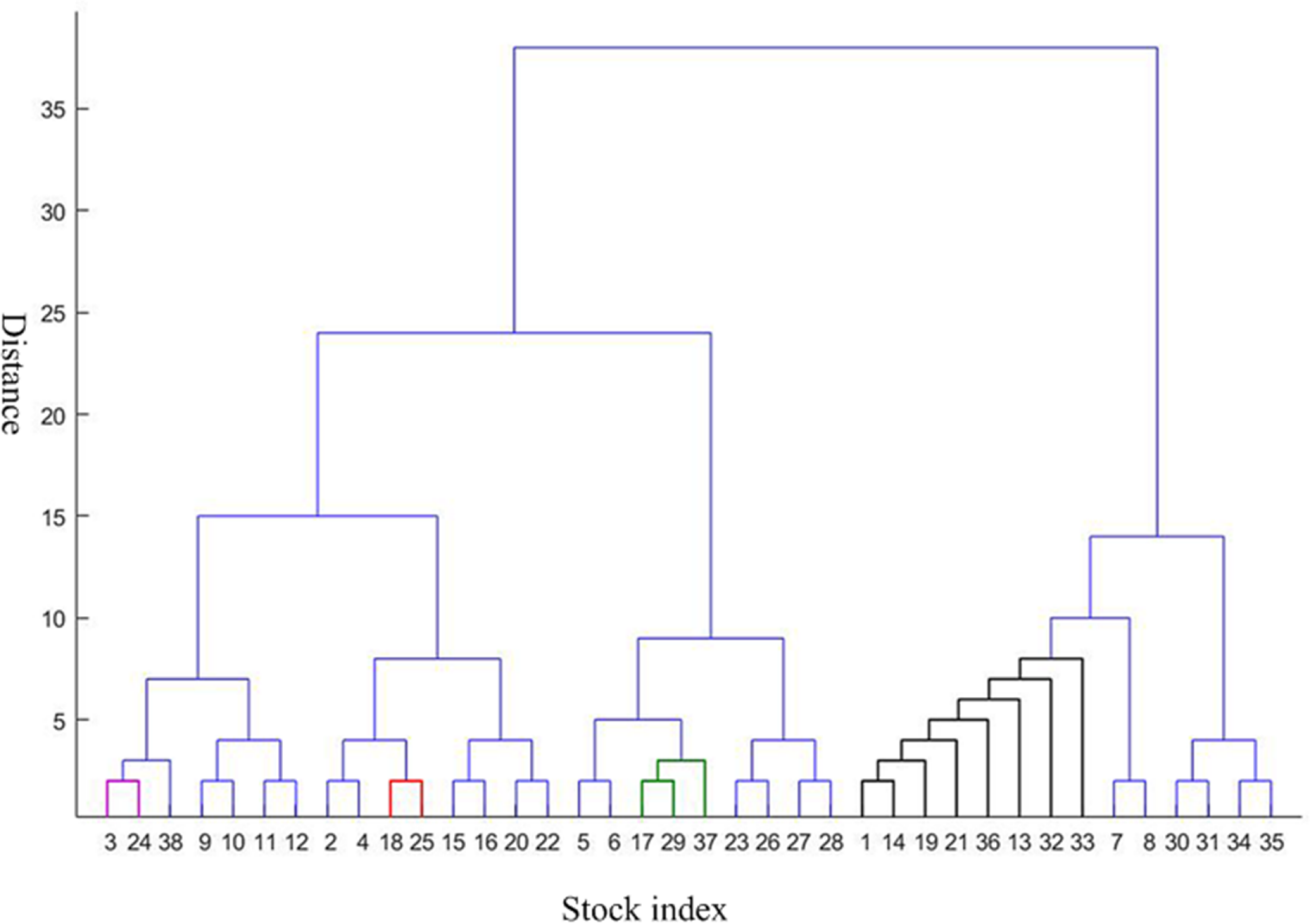
Hierarchical clustering structure after the crisis. This is the hierarchical clustering structure after the crisis. The horizontal axis represents the stock index, while the vertical axis represents the distance between these indices. The network could be divided into four communities whose member is shown in purple, red, green and black line.

To further analyze the community structure of the network before and after the crisis in this threshold, in this paper, the method proposed by Newman ME [32] is used to obtain hierarchical tree diagrams, which could find the community structure and clustering effect in the network. When the threshold is 0.9, the network before the crisis contains 14 nodes and 18 edges. Other 24 nodes are isolated nodes. The peak modularity is Q = 0.3210. The network could be divided into four communities which is shown as Fig 9 in purple, red, green and black line. This indicates a strong linkage among them in each community. The first community consists of 3 and 24. It is the index of Shanghai Composite Index and Shenzhen Component Index in China. The second communities are 18 and 25, which is S & P Australia 200 Index and Australian Common Stock Index in Australia. The third communities include 17, 29, and 37. They are S & P 500 index, NASDAQ Composite Index and Dow Jones Industrial Average index respectively. It is not difficult to observe that the above three communities are come from the same country and region, which are closely related to them. This is consistent with the correlation coefficient between them in above Figs 2–4. The fourth communities are 1, 32, 19, 36, 13, 21, 33. They represent FTSE Europe Pioneer 300 Index, UK FTSE 100 Index, European Stoxx 600 index, Spain IBEX35 index, German DAX30 index, France CAC40 index and Netherlands Amsterdam Index, which are all from the European market. It may be related to the European Union countries. For the network after the crisis contains 15 nodes and 27 edges. Other 23 nodes are isolated nodes. The peak modularity is Q = 0.3765. Similar to above Fig 9, the community result is shown in Fig 10. The composition of the four community structures is similar to that in the stage of before the crisis. This is no longer repeated here.

## Conclusion

In this paper, we presented a detailed analysis of global stock index data of major countries in the world before, during, and after the 2008 financial crisis, by means of complex network method. New analysis and discovery of network characteristics and rules give us a comprehensive perspective for global stock market during the crisis. It would be helpful to investors for making decisions regarding their portfolios or to administrators for controlling stock market. Therefore, this research is useful from both theoretical and practical points of view.

For three weighted networks, the physics-derived technique of minimum spanning tree is utilized to investigate the networks of three stages. It indicates that all the network has clustering phenomenon. Three cluster structures were found, which are from the American stock market, Southeast Asia stock market and European stock market. Besides, core nodes after the crisis has been weakened with edge number reduced. At last, the change of weight between stock indexes is not balanced. Such the clustering structure could be monitored by the regulators, in order to ensure the overall stability of the global stock market.

For three threshold networks, we find that the average threshold network before and during the crisis has shown the clear small-world property. Besides, a comprehensive analysis of the three periods of network, the property of the gradual weakening trend. We also further deeply analyze the change of community structure of threshold networks with different thresholds. The result indicates that for large thresholds, the network before and after the crisis has a significant community structure, and the network during crisis is the opposite. Such a stability link in community would be also useful for portfolio investments and risk management.

Although we addressed many issues in our analysis of the global stock index network, there are still a lot of open problems. We could conduct the analysis on rolling windows of different sizes in a temporally dynamic network. It is also possible to explore an early warning indicator of a crisis based on network properties, taking the stock index and external economic indicators into account. This will be an important research direction in our future work.

## Supporting information

S1 File Global stock index data 2005–2010. See the respective attached file.
(ZIP)

S2 File The mapping from numbers to the names of indices. (ZIP)

S3 File The value of correlation coefficient matrix before, during and after the crisis for Figs 2–4. (ZIP)
